# Estimating the Effects of Public Health Measures by SEIR(MH) Model of COVID-19 Epidemic in Local Geographic Areas

**DOI:** 10.3389/fpubh.2021.728525

**Published:** 2022-01-04

**Authors:** Tianyi Qiu, Han Xiao, Vladimir Brusic

**Affiliations:** ^1^Shanghai Public Health Clinical Center, Fudan University, Shanghai, China; ^2^Department of Computer Science, Aalto University, Espoo, Finland; ^3^School of Computer Science, University of Nottingham Ningbo China, Ningbo, China

**Keywords:** mathematical model, public health policies, lockdown, hospital capacity, COVID-19

## Abstract

The COVID-19 pandemic of 2020–21 has been a major challenge to public health systems worldwide. Mathematical models of epidemic are useful tools for assessment of the situation and for providing decision-making support for relevant authorities. We developed and implemented SEIR(MH) model that extends the conventional SEIR model with parameters that define public lockdown (the level and start of lockdown) and the medical system capacity to contain patients. Comparative modeling of four regions in Europe that have similar population sizes and age structures, but different public health systems, was performed: Baden-Württemberg, Lombardy, Belgium, and Switzerland. Modeling suggests that the most effective measure for controlling epidemic is early lockdown (exponential effect), followed by the number of available hospital beds (linear effect if the capacity is insufficient, with diminishing returns when the capacity is sufficient). Dynamic management of lockdown levels is likely to produce better outcomes than strict lockdown.

## Introduction

Mathematical models of epidemic help predict the spread of infection and identify the likely outcomes of an epidemic (1, 2). These models provide information about the likely effects of public health interventions enacted to control the epidemic. Epidemiological models provide support for decision making related to early intervention or ending the measures. Imposing effective and timely measures is essential for the disruption of the rapid spread stage of epidemics (3). Compartmental epidemiological models assign population to compartments labeled by their health status. For example, the SEIR model assigns population to **S**usceptible, **E**xposed, **I**nfectious, and **R**ecovered subpopulation compartments (4, 5). These models are used to predict epidemiological parameters, such as disease spread, the total number of infections, and the shape of epidemiological curves (6–8). SEIR models have been used for modeling epidemics caused by influenza virus (9), Ebola virus (10), Middle East respiratory syndrome-related coronavirus (MERS-CoV) (11), and human immunodeficiency virus (HIV) (12).

In the past, actual epidemiological data were available only with a delay. Earlier models could only assess the dynamics of the outbreak and the effects of control measures after the outbreak (1, 13). The post-epidemic models focused on modeling the natural spread of infection and usually did not include the intervention measures as part of the model. Rather, the interventions were considered as the means to change the basic epidemic parameters directly. Advances in information and communication technologies have enabled an unprecedented speed of data exchange, and the updates of basic epidemiological parameters are now available daily (5). Timely updates enable building of modified SEIR models that incorporate public health measures as internal model parameters. This, in turn, enables the adjustment of basic SEIR models, as observed during the COVID-19 pandemic (6, 8, 14). The modifications include the addition of relevant parameters, such as migration index (15), speed of the infection during latent period (16), asymptomatic carriers' populations and personal intervention strategy (17, 18), simulation of the final phase of the outbreak (19), or seasonality (20). These adjustable models were developed using early data from specific limited locations and are based on assumptions that were not yet confirmed at the time of modeling. The common theme with these models is that they are reasonable approximations of actual epidemic spread. Most of these models represent extensions of the basic model, for example, the SIDARTHE model (14) defines eight population compartments that provide additional insight about populations at risk. Our extension of the basic SEIR model considers key public health variables and their combined effect on the control of epidemic.

We developed a modified SEIR model, SEIR(MH), that includes additional modeling parameters as compared to the base model. These additional parameters include the capacity of the public health system to support control measures, such as the conditions of public lockdown (level of lockdown F, and the start date of lockdown T_L_), and the available capacity of the medical system to contain patients (the population with access to healthcare M, and the number of dedicated hospital beds H). The SEIR(MH) model was applied to the COVID-19 data from four regions in Europe that are comparable by population sizes and socio-economic status: Baden-Württemberg (Germany), Lombardy (Italy), Belgium, and Switzerland. These four regions represent a variety of lockdown conditions and different initial capacities of the medical system to contain the spread of infection. The results of simulations by the SEIR(MH) model agreed well with the observed curves of daily epidemic reports. Using these data and the SEIR(MH) model, we estimated the actual COVID-19 epidemic progression in these four regions during the first wave. We used the resulting models to analyze what-if scenarios to study the effects of different lockdown policies and the numbers of COVID-19 available beds, using the real reports data. Finally, we performed simulations of COVID-19 epidemic situations in three virtual cities with different age structures to demonstrate the potential utility of the SEIR(MH) modeling for designing optimized public health measures.

## Materials and Methods

### Data Sources and Assumptions

The daily statistics of new infections, current infections, and fatalities in four studied regions (Baden-Württemberg, Belgium, Lombardy, and Switzerland) were obtained from the COVID-19 projections of IHME (21). In this resource, the migration index before and after the lockdown was also collected, as well as the COVID-19 available beds (Supplementary Table 1) (21). These data have been updated daily through concerted effort of many individuals and organizations and rapidly shared with the community.

We added two state variables including *M* and *H* to extend the traditional SEIR model (5, 15). Variables *S*, *E*, *I*, *R*, and *M* represent the total number of people in each corresponding state. *S*, *E*, *I*, and *R* represent the number of susceptible, exposed (infected without symptoms), infected with symptoms, and removed individuals, respectively. *M* represents the number of people with medical care and *H* represents COVID-19 available beds in hospitals (22). Current number of infections is defined as *I*+*M* and the total number of infected individuals as *I*+*M*+*R*. For modeling, we made the following assumptions:

- People in state *S* will transit to state *E* after infection and cannot transit to state *I* directly.
- All people in state *E* will eventually transit to state *I* after the incubation period.
- People in state *I* will transit to state *M* when beds are sufficient, or transit to state *R* when self-recovered or died, without medical care.
- People in state *M* will transit to state *R* when recovered or died. We did not consider the possibility of re-infection in the current model.
- People in states *E* and *I* are infectious with the infection coefficients of α and β, people in state *M* are not infectious. α and β are mobility-related parameters.
- The number of individuals in state *M* should be less than or equal to the total number of *H* at each time point.
- The incubation period follows Poisson distribution with the mean time between 4 and 7 days (23).
- The COVID-19 available beds indicate the capacity of hospitals to take in COVID-19 patients.
- The probability of people to transit from *I* to *M* is a function of the current number of beds and waiting time for hospitalization. The more available beds and the longer the waiting time, the higher the probability. The conversion probability for each day follows the sigmoid function with the mean waiting time between 4 and 8 days (24).
- The hospitalization time follows Poisson distribution with the median time between 7 and 14 days (24).

### Model Specification

The SEIR(MH) model is a state recursive model, where the estimated values of key model parameters were calculated using recursive formulas based on daily simulation of epidemic. Model inputs were the reported data from previous days. For SEIR(MH) modeling, the epidemic is divided into evenly spaced time steps measured in days. The size of SEIR(MH) population in each time step is described by the corresponding states: *Susceptible* (S), *Exposed without symptoms* (E), *Infected with symptoms* (I), *Removed from the system* (R), *with medical care* (M), and *the maximum number of beds in hospitals* (H).

We use symbols *X* and *Y* for state transitions. The uppercase *T* is used for the absolute date and lowercase *t* is used for the offset in days (e.g., *t* = 3 or −3 mean “3 days later” or “3 days earlier”). For any day *T* and state *X*, *X*(*T*) stands for the size of population in state *X* on day *T*. For instance, *I*(*T*) is the number of symptomatic infections on day *T*. The epidemic states on each day *T* are represented by {*X*(*T*) | *X*∈{*S, E, I, R, M, H*}}.

**Figure 1:**
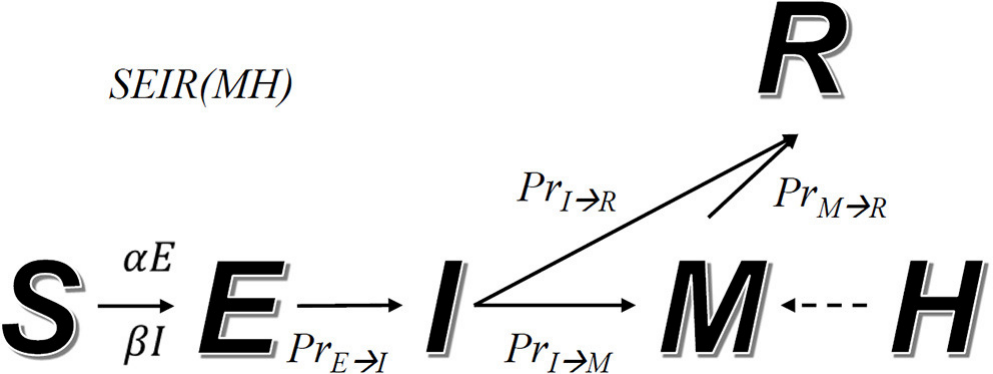
Transition diagram of the SEIR(MH) model. S, E, I, M, and R represent individuals in states: susceptible, exposed without symptoms, infected with symptoms, with medical care, and removed from the system. The number M cannot be larger than the current number of beds in hospitals, defined as H. The arrow indicates the direction of state transmission. The infection coefficients determine transition numbers S to E (α) and S to I (β). *Pr*_*E*→*I*_, *Pr*_*I*→*R*_, *Pr*_*I*→*M*_ and *Pr*_*M*→*R*_ represent transmission probability between indicated states.

During an epidemic, we assume that a fraction of the population will transit from one state to another. For instance, a fraction of the population infected with symptoms (state *I*) starts to receive medical care (changing to state *M*). The relations among these states are defined by a transition diagram, shown in Figure 1. Among the variables, *S, E, I, R*, and *M* make direct transitions, while the value of *H* is the upper bound of *M*, meaning that hospitals cannot receive more patients than their capacity.

Our model simulates the progression of an epidemic. The transition rules (i.e., how many people will transit from one state to another at any time step) are defined by a set of formulas and adjustable parameters. Given two different states *X* and *Y*, *Pr*_*X*→*Y*_(*t*) represents the probability for a person in state *X* to transit to state *Y* after *t* days. According to the state transition diagram, we have the following functions: *Pr*_*E*→*I*_(*t*), *Pr*_*I*→*R*_(*t*), and *Pr*_*M*→*R*_(*t*). An exception is *Pr*_*I*→*M*_(*t*|*T*−*t*) which depends on both *T* and *T*−*t*. This is related to the fact that *M*(*T*) (number of people in hospital) has upper bound *H*(*T*) (hospital capacity), which changes over time (due to addition of hospital beds). Other parameters include infection coefficients α and β that control how fast individuals in states *E* and *I* infect unexposed individuals. α and β are constant when interventions remain unchanged or no intervention is taken.

Δ*X*(*T*) = *X*(*T*)−*X*(*T*−1) is the difference between population sizes in state *X* on days *T* and *T*−1. Positive Δ*X*(*T*) indicates the growth of population *X* between *T*−1 and *T*, zero indicates stable situation, while negative number indicates decline. In our simulation, the values of Δ*E*(*T*), Δ*I*(*T*), Δ*M*(*T*), Δ*H*(*T*), and Δ*R*(*T*) are calculated for each day *T*. By definition *X*(*T*) = *X*(0)+ Δ*X*(1)+…+Δ*X*(*T*) for any state *X*, where *X*(0) as the initial input. Thus, we can simulate the status for any day *T* and *X* by calculating *X*(*T*). The patient zero (index case) *E*(0) and the day of patient zero *T*_0_ are not the actual cases because the initial infection is usually a cluster of cases imported from outside. The *E*(0) and *T*_0_ are an idealized case where there is a virtual patient on a particular day that would produce the same infection dynamics as the imported cluster of cases.

We defined Δ^+^*X*(*T*) as transit population from the preceding state to state *X*, which must be non-negative. For instance, when the exposed with symptoms (*E*) are 0, no people will transit to the infected state (*I*) and Δ^+^*X*(*T*) = 0. ${\text{Δ}}^{+}\text{X(T }-{\text{t)Pr}}_{\text{X}\to \text{Y}}\text{(t)}$ represents the number of people who enter the state *X* on day *T*−*t* and then change to state *Y* on day t (after *t* days). For instance, ${\text{Δ}}^{+}\text{E(T }-{\text{1)Pr}}_{\text{E}\to \text{I}}\text{(1)}$ is the number of individuals who became infected with symptoms (enter state *E*) on day *T*−1 and start to show symptoms on the next day (enter state *I*). If Δ*E*(*T*−1) < 0, ${\text{Δ}}^{+}\text{E(T }-{\text{1)Pr}}_{\text{E}\to \text{I}}\text{(1)= 0}$.

### Transition Rules

The definition of the symbols Δ*X*(*T*) for *X*∈{*S, E, I, R, M, H*} as well as transition probability *Pr*_*X*→*Y*_(*t*) are defined as:

**Δ***E*(**T**) consists of two components corresponding to two scenarios: (1) susceptible population (in *S*) become infected (enter state *E*), (2) infected population (in *E*) start to show symptoms (enter state *I*). For *S* to *E* transition, min{1, *E*(*T*−1)α+*I*(*T*−1)β} was defined for the overall infection probability due to contact with populations *E* and *I*. We added the min operator to ensure the probability cannot exceed 1. Multiplying this probability by *S*(*T*) leads to the increase of the population in state-*E*. For *E* to *I* transition, ${\text{Δ}}^{+}\text{E(T}-{\text{t)Pr}}_{\text{E}\to \text{I}}\text{(t)}$ equals to the number of people who become infected on *T*−*t* and start showing symptoms *t* days later. For simulations we define a time span of *k* days. Thus, a total number of $\sum_{t=1}^{k}{\text{Δ}}^{+}\text{E}\left(\text{T}-\text{t}\right){\text{Pr}}_{\text{E}\to \text{I}}\left(\text{t}\right)$ that transit from *E* to *I* are:


(1) $$\begin{array}{l} \Delta E\left(T\right)=S\left(T\right)\left(\;E\left(T-1\right)\alpha +I\left(T-1\right)\beta \right)\\ \;-\sum_{t=1}^{k}E\left(T-t\right){\mathrm{Pr}}_{E\to I}\left(t\right) \end{array}$$

Δ***I(T)*** has three components: (1) infected people (in *E*) start showing symptoms (enter state *I*), (2) people with symptoms (in *I*) start to receive treatment in hospital (enter state *M*), and (3) people with symptoms (in *I*) die or recover (enter state *R*). The first component maps to the last term in equation (1). The last two components are defined as:


(2) $$\begin{array}{l} \Delta I\left(T\right)=\sum_{t=1}^{k}\Delta^{+}E\left(T-t\right)Pr_{E\to I}\left(t\right)\\ \;-\sum_{t=1}^{k}\Delta^{+}I\left(T-t\right)Pr_{I\to M}\left(t|T-t\right)\\ \;-\sum_{t=1}^{k}\Delta^{+}I\left(T-t\right)Pr_{I\to R}\left(t\right) \end{array}$$

Δ***M(T)*** captures the following scenarios: (1) patients under medical treatment (in state *M*) can recover or die (enter state *R*), (2) population of infected individuals (in status *I*) can be admitted to hospital (in state *M*), and (3) the number of new admissions to hospitals must not exceed the hospital capacity. The Δ*M*(*T*) captures three scenarios:


(3) $$\begin{array}{l} \Delta M\left(T\right)=min[\sum_{t=1}^{k}\Delta^{+}I\left(T-t\right)Pr_{I\to M}\left(t|T-t\right)\\ \;-\sum_{t=1}^{k}\Delta^{+}M\left(T-t\right)Pr_{M\to R}\left(t\right),\\ \;H\left(T-1\right)-M\left(T-1\right)] \end{array}$$

Δ***R(T)*** captures the population that entered state *R* from either state *I* or *M*, which are both non-positive:


(4) $$\begin{array}{l} \Delta R\left(T\right)=\sum_{t=1}^{k}[\Delta^{+}I\left(T-t\right)Pr_{I\to R}\left(t\right)\\ \;+\Delta^{+}M\left(T-t\right)Pr_{M\to R}\left(t\right)] \end{array}$$

**Pr**_**E**→**I**_. We assume *Pr*_*E*→*I*_(*t*) follows Poisson distribution where λ_*E*→*I*_ is the average number of days for infected individuals to start showing symptoms:


(5) $$Pr_{E\to I}\left(t\right)=Poission\;\left(t;\;\lambda_{E\to I}\right)$$

**Pr**_**I**→**M**_. *Pr*_*I*→*M*_(*t*|*T*−*t*) is the probability of being admitted to a hospital after *t* days since day *T*. We assume *Pr*_*I*→*M*_(*t*|*T*−*t*) follows geometric distribution where *P*_*T*−*t*_ is the probability that an infected individual is admitted to hospital precisely on the day *T*−*t*. During the time of an epidemic, people who need hospitalization may not be admitted if the hospital capacity is full, so placement in another hospital will be requested. The larger the number of the patients already in hospital, the more difficult it is for newly diagnosed patients to find a place in the hospital. This situation is well-represented by geometric distribution:


(6) $$Pr_{I\to M}\left(t|T-t\right)=P_{T}\cdot \prod_{t=1}^{T}\left(1-P_{T-t}\right)$$

*P*_*T*_ is a variant of logistic function:


(7) $$P_{T}=\{\begin{array}{c} \frac{1}{1+\exp(-k\left(H\left(T\right)-M\left(T\right)-x_{0}\right))},\;H\left(T\right)-M\left(T\right)>0\\ 0,\;otherwise \end{array}$$

where *k* and *x*_0_ are model parameters to be fitted according to the waiting time for people to receive medical care. *P*_*T*_> 0 when the hospital capacity for COVID-19 is not reached, *H*(*T*)−*M*(*T*)> 0.

**Pr**_**I**→**R**_. We assume that infected population either recovers or dies (enter *R*) if they are not admitted to hospital after *t*_*x*_ days. *t*_*x*_ is a model parameter to be fitted and *Pr*_*I*→*R*_(*t*) is defined as:


(8) $$Pr_{I\to R}\left(t\right)=\{\begin{array}{c} >t_{x},\;1\\ 0,\;otherwise \end{array}$$

*Pr*_*M*→*R*_. We assume that *Pr*_*M*→*R*_(*T*) follows Poisson distribution where λ_*M*→*R*_ is the average number of days for transition from *M* to *R*, e.g., average days to recovery or before death under medical care:


(9) $$Pr_{M\to R}\left(T\right)=Poisson\left(T;\lambda_{M\to R}\right)$$

### Model Parameters Fitting

The fitting objective was to minimize the mean absolute error between the predicted number of increased infections (*I*) and the observed number of increased infections. Since SEIR(MH) is a state recursive model, the estimated number of increased infections (*I*) at time *T* and the estimated number of increased exposed population (ΔE) at time *T* do not have a closed-form solution for given sets of parameters. The estimated values of (ΔI) and (ΔE) could not be obtained by parameter estimation but were assessed by exploration of the search space. Thus, model optimization is a non-trivial task. To ensure that the solution space is fully explored, we resorted to brute-force search over the pre-specified ranges of parameters. To reduce computation overhead, the range of each parameter was discretized into evenly spaced values. In addition, we performed search of the optimized parameters in two steps: (1) narrowed down the searching space in the first round and (2) refined to a greater precision in the second round.

The infection coefficients before the lockdown are α_pre_ and β_pre_ and after the lockdown are α and β. The infection coefficients α and β were estimated for each region by model fitting to the reported data. Lockdowns cause changes of the mobility factor (parameter F). In our model F ranges from 1 (no reduction of mobility) to 6 (extremely high reduction of mobility). The corresponding infection coefficients before the lockdown were calculated by α_pre_ = α × F and β_pre_ = β × F.

### Simulation of Virtual Cities

The overall goal of virtual city epidemic simulations was to help identify the optimal level of public health measures given three variables: the lockdown date, lockdown level, and the number of beds. The lockdown date T_SDn_ is time in days from the estimated patient zero day (T_SD0_). The earliest lockdown date in simulation was T_SD24_, 24 days from T_SD0_. The latest lockdown date in simulation was T_SD72_, 72 days from T_SD0_. In the first stage, we fixed the number of COVID-19 beds (four per thousands) and performed the simulation analysis of TNI and TND for three virtual cities. Two parameters were varied in this simulation: days between the first patient to lockdown date (T_SDn_) and the lockdown factor. The second stage of simulation involved the same procedure as in stage 1, but for eight additional available COVID-19 bed values (0.5 to 4, increment 0.5). The third stage involved systematic changes of all three variables to identify optimal lockdown level for each lockdown start date (24 to 72, increment 2) and three levels of the number of beds (1, 2, and 4).

The total number of infected people (TNI) on any day during the epidemic was defined as the total number of people in status *I*, status *M*, and status *R*. The COVID-19 infection rate (*CIR*) at each day equals the ratio *TNI*/*TPO* where *TPO* is the total population of the region. Using the SEIR(MH) modeling, the CIR on each day of the three virtual cities was calculated. The overall death rate in a population (*PDR*) was calculated as *PDR* = *CIR*×*CDR*, and the total number of COVID-19 deaths was calculated as *TND* = *CIR*×*CDR*×*TPO*. Using the simulation, we could estimate the effects of public health strategies (lockdown time, lockdown level, and available beds for COVID-19) for regions with different age structures using the overall number of deaths in each region (*TND*) as the minimization target. The simulation variables for cities are available in Supplementary Table 2. The first stage had a total of 2,100 simulations, the second stage involved an additional 4,200 simulations, and the third stage involved 4,725 simulations. All simulations used a quasi-exhaustive search (25) to find the best level of lockdown for possible situations arising from combinations of the lockdown date, number of beds, and the age structure.

## Results

### Estimation of Parameters of the SEIR(MH) Model

In our model, we divided the epidemic progress into discrete periods, measured in days. The model has four population variables and two health system capacity variables. The population variables provide the size of epidemiology categories (or compartments): *Susceptible* (S), *Exposed without symptoms* (E), *Infected with symptoms* (I), and *Removed from the system* (R). The health system capacity variables include the *population with medical care* (M) and the *maximum number of beds in hospitals* (H). All of these variables are time dependent. Among them, the values of *S*, *E*, *I*, *R*, and *M* at each date are simulated, and the values of *H* are pre-specified.

The SEIR(MH) model contains eight epidemiological parameters including infection coefficients α and β; transition times *T*(*E*→*I*), *T*(*I*→*M*), *T*(*I*→*R*), *T*(*M*→*R*); the mobility change factor *F*; the region-specific constant *O*(*T*); and region-specific time shift variable ΔT. In this study, the SEIR(MH) model was generated using data from four European regions including Baden-Württemberg, Lombardy, Belgium, and Switzerland. These regions have comparable population sizes, ranging from 8.57 to 11.48 million, and a similar age distribution of population (Supplementary Table 3). The values of model parameters in these four regions, their descriptions, and how they are determined are shown in Table 1. Among them, the values of α, β, *T*(*I*→*R*), *F*, and *O*(*T*) were fitted to the model using data from daily reports of the new infections in studied regions. The values of *T*(*E*→*I*), *T*(*I*→*M*), and *T*(*M*→*R*) were pre-specified using data from published research and available reports. A detailed description of parameter fitting is described in the ***Materials and Methods*** section.

**Table 1:** Parameters used in SEIR(MH) modeling.

**Variable**	**Variable meaning**	**BW** ^b^	**Bel** ^c^	**Lom** ^d^	**Swi** ^e^	**Estimation**
α^a^	Infection coefficient by people at state E	1.1 ×10^−8^	1.1 ×10^−8^	1.2 ×10^−8^	1.3 ×10^−8^	Data fitting
β^a^	Infection coefficient by people at state I	1.8 ×10^−9^	1.1 ×10^−9^	1.4 ×10^−9^	1.3 ×10^−9^	Data fitting
T(E->I)	Average days for people converting from E to I	6	6	6	6	PMID^f^: 31995857^1^
T(I->M)	Average days for people converting from I to M	7	7	7	7	PMID^f^: 32031570^7^
T(I->R)	Average days for people converting from I to R	14	14	14	14	Data fitting^g^
T(M->R)	Average days for people converting from M to R	10	10	10	10	PMID^f^: 32031570^7^
F	Mobility change due to lockdown	3.00	3.47	3.85	2.55	Data fitting
O(T)	Days between first patient to lockdown	39	31	31	59	Data fitting
ΔT^h^	Shift curve to earlier date	0	0	−7	0	Adjustment

a *α and β are the infection coefficients after the lockdown. The corresponding infection coefficients before the lockdown are calculated as α_pre_ = α × F and β_pre_ = β × F. The infection coefficient before lockdown is associated with the mobility. The infection coefficients were estimated for each region by data fitting, considering changes of the factor of mobility after the lockdown (parameter F). In our model it ranges from 1 (no reduction of mobility) to 6 (extremely high reduction of mobility)*.

b *Baden-Württemberg in Germany (BW)*,

c *Belgium (Bel)*,

d *Lombardy in Italy (Lom)*,

e *Switzerland (Swi)*.

f *Pubmed ID*.

g *Considering it is a disease-related intrinsic parameter, we assume the T(I->R) in each region is equal*.

h *Early outbreaks are associated with delayed reporting of cases, lack of testing kits, and difficulties in identification true cases*.

### Agreement of Fitted Models With the Reported Data From the Studied Regions

**Figure 2:**
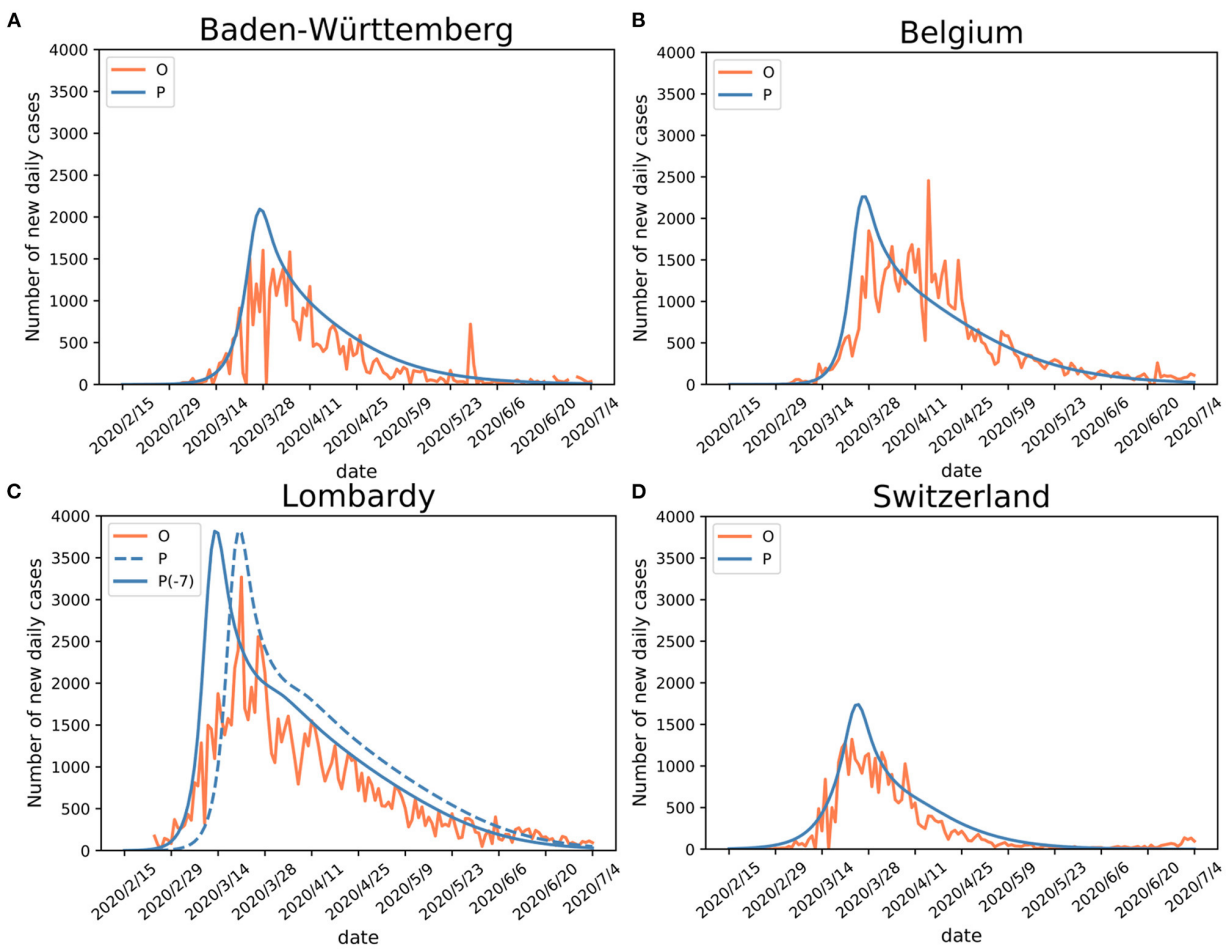
Validation of SEIR(MH) model. Comparisons of the Infected with symptoms (I) population (observed “O” and estimated “P”) curves in **(A)** Baden-Württemberg, **(B)** Belgium, **(C)** Lombardy (“P-7” stands for adjusted curve), and **(D)** Switzerland. Shifting the curve to the earlier days (Δ*T* = −7) shows better matching with the observed data during the early epidemic period in Lombardy. The justification for Lombardy model adjustment is given in the main text.

The SEIR(MH) model was evaluated using observed data from the four European regions (Table 2). These regions differed in their public health policies and utilization of medical resources during the first onset of the COVID-19 pandemic. For each region, the number of active infections was used as the indicator for model evaluation. Using the mobility data derived from COVID-19 projections of the Institute for Health Metrics and Evaluation (IHME) (21), we defined two distinct stages of the early period of the epidemic: initial stage (without movement restrictions) and public measure stage (with movement restrictions—the lockdown). The performance of the SEIR(MH) model in comparison to the actual reported observations is shown in Figure 2.

**Table 2:** Estimated model parameters and observed values in four studied regions.

**Region**	**Baden-württemberg**	**Belgium**	**Lombardy**	**Switzerland**
Period^a^	February 15–July 4, 2020			
r^b^	0.83	0.84	0.88	0.93
Days	140			
OPT^c^	March 28	March 28 (true)/April 15 (outlier)	March 21	March 23
OPI^d^	1,603	1,850/2,454	3,268	1,321
EPT^e^	March 27	March 27	March 20	March 25
EPI^f^	2,093	2,259	3,815	1,739
S^g^	11.07	11.48	9.95	8.57
oTNI^h^	36,275	61,837	94,318	32,198
eTNI^h^	54,494	67,239	127,899	42,848
eTNI^i^	54,646	67,691	128,584	42,892

a *Calendar periods of SEIR(MH) modeling for each region*.

b *Correlation coefficient between observed and estimated infections (r)*.

c *Observed peak time (OPT)*.

d *Observed peak infections (OPI)*.

e *Estimated peak time (EPT)*.

f *Estimated peak infections (EPI)*.

g *Total population (millions) in each of the four regions (source: data.worldbank.org)*,

h *Total number of observed (oTNI) and estimated (eTNI) infections till July 4, 2020*.

i *Total number of estimated (eTNI) infections till estimated end point*.

For Baden-Württemberg, the correlation coefficient between the observed and estimated daily active infections (population *I*) was *r* = 0.83 from February 15 to July 4. The observed peak of daily new infections happened between March 24 and April 5. There were three days with the values of *I* number larger than 1,500 (March 24, March 28, and April 5). The infection peak estimated by the model occurred on March 27, with 2,093 people estimated to be in status *I* (Figure 2A; Table 2).

For Belgium, the correlation coefficient between observed and estimated daily active infections was *r* = 0.84. The first observed peak appeared on March 28, with 1,850 infections, in agreement with the estimated peak on March 27, with 2,259 estimated infections in status *I* for both days (Figure 2B). Belgium had a second peak observed on April 15, with 2,454 infections. This peak appears to be a reporting artifact (only ~500 infections were reported on the previous day).

The comparison of observed and estimated populations *I* for Lombardy-Italy (*r* = 0.88) and Switzerland (*r* = 0.93) showed good agreements between the observed and estimated numbers of active infections (Figures 2C,D). The detailed information is shown in Table 2. The number of newly reported infections can substantially differ from the actual number of new cases due to reporting and testing delays (26). After considering these delays, our model shows consistency between the estimated infection curves (theoretical expectations) and the observed reported cases. Our modeling results show similar patterns of infection curves in the four regions. The main difference between the overall estimated infection curves is the flatness of the infection curves. The infection rates in Belgium and Switzerland showed slower decline of the infection curve than those in Baden-Württemberg and Lombardy during the middle of the epidemic period. The comparison of estimated infection curves to the actual data indicated that the SEIR(MH) of the COVID-19 epidemic is consistent with the actual epidemiological situation observed in these four regions during the epidemic.

The infection curve for Lombardy shows a poor match between observed and estimated (by model) infections during the earliest stage of the infection spread. The analysis of Lombardy data suggested that the reporting of the actual number of infections was delayed by up to 7 days. The evidence for delayed reporting includes (1) the first reporting on February 23 was a cluster of cases rather than the individual index case (https://www.ecdc.europa.eu/en). (2) On February 14 a 38-year-old Italian in Lombardy felt unwell and visited a doctor. The patient was prescribed treatments for influenza but was later confirmed as a COVID-19 case (as reported in Italian and Swiss newspapers). Thus, the adjusted curve for Lombardy was moved to 7 days earlier to accommodate the initial delay (shown in Figure 2C).

### Dynamic Modeling of the Epidemic in Four European Regions

**Figure 3:**
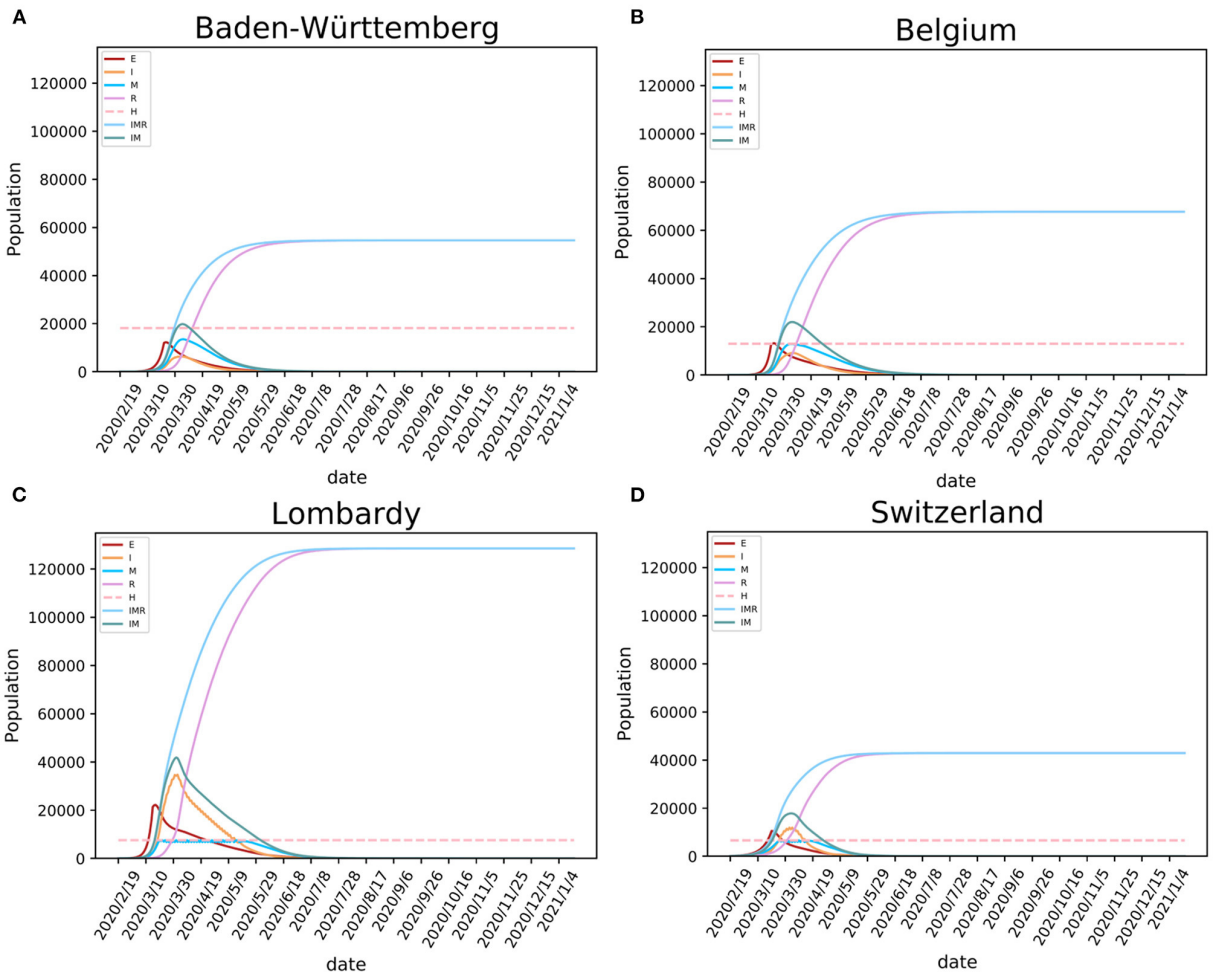
Modeling the SARS-CoV-2 epidemic in four European regions. The epidemic models of SARS-CoV-2 are shown for **(A)** Baden-Württemberg (Germany), **(B)** Belgium, **(C)** Lombardy (Italy), and **(D)** Switzerland. E, I, and R stand for exposed, infected, and recovered population, M for population having medical care in hospitals (occupancy of beds), IMR stands for I+M+R, and IM stands for I+M (active cases).

Using fitted parameters, the epidemic dynamic in four studied European regions was estimated using the SEIR(MH) modeling. For each region, the epidemic was divided into two periods. The time from the first patient to the date which the mobility reduced to the minimum level is considered as the period before lockdown. The other period covers time after the lockdown. The populations with the daily status of *S*, *E*, *I*, *R*, and *M* at each specific date were estimated by the model. The values of *H* are public health statistics data that were obtained from the COVID-19 projections at IHME (21). For Baden-Württemberg, the first period was from February 12 to March 22, 2020. During that period weaker public health interventions were applied, and the number of infections increased rapidly (Figure 3A). After the lockdown, the mobility of people decreased sharply. We estimated that the population sizes representing states *E*, *I*, and *M* on March 22, 2020, were 11,977, 1,709, and 2,530, respectively. The model showed 4,239 infected individuals with symptoms (people in states *I* and *M*), similar to the reported number of 3,768 on the same date. However, our model estimated 11,977 individuals that were exposed but without symptoms who could also be infectious. After the lockdown, the daily infections quickly reached the peak and then started dropping rapidly (Figure 3A). The shapes of the curves indicate the benefit from the decrease of mobility following the lockdown. The epidemic estimations for Belgium, Lombardy, and Switzerland (Figures 3B–D) show similar shapes of infection curves to Baden-Württemberg: rapid increase before the lockdown followed by sharp decrease after the peak.

Our model showed that the need for hospital beds (curve *M*) is always lower than the available beds for COVID-19 (curve *H*) in Baden-Württemberg and Belgium (Figure 3). These numbers indicate that there was sufficient hospital capacity for COVID-19 patients. In Lombardy and Switzerland, bed numbers were lower than needed during the early period of the epidemic. In Switzerland, this period lasted for 18 days, from March 26 to April 12. During this period, over 14,518 infections were observed, 806 infections per day on average. This created a significant requirement for new beds designated for COVID-19, given the initially available number of 6,596 (21). In Lombardy, this period lasted for 58 days, from March 20 to May 16. In addition to beds designated for COVID-19, the four regions had additional medical resources that could provide a total number of 3.2–8 beds per thousand population (https://data.worldbank.org/indicator/SH.MED.BEDS.ZS) The COVID-19 pressure on limited medical resources precipitated government intervention of strict lockdown, such as one enforced in Lombardy.

Modeling results suggest that, if the regions kept strict lockdown policies, the first wave of the epidemic in Baden-Württemberg would end by August 31 with 54,646 total infections. Under conditions of prolonged lockdown, our model suggests that the end of the first wave would happen by September 24, 2020, in Belgium with 67,691 total infections, by September 17 in Lombardy with 128,584 total infections, and by July 24 in Switzerland with 42,892 total infections (Table 2). These results must be considered with caution because ideal situations are difficult to implement in a real-world situation. Due to socioeconomic issues and other cost of lockdowns, it may be difficult to maintain the lockdown for a long time. The release of lockdown and the influx of imported cases may lead to subsequent waves of the epidemic. The end of the epidemic would come earliest as the result of the lockdown, while the total number of infections would also be relatively low given that sufficiently large numbers of hospital beds were available, but the population will not develop useful levels of herd immunity. In reality, the epidemic in these regions did enter the second wave that has shown different dynamics (larger number of infections and lower mortality rate) than the first wave. Considering that studied regions implemented different policies of lockdown and had different initial resource availability, this case study provides means to study the consequences of the date of lockdown, the level of lockdown, and the number of available hospital beds for control of COVID-19 spread (Table 3).

**Table 3:** The lockdown levels and available beds of four regions.

**Region**	**Highest mobility^a^**	**Lowest mobility^b^**	**Lockdown level^c^**	**Beds available^d^**
Baden-württemberg	14.10	−55.54	69.65	18,114
Belgium	3.44	−77.61	81.05	12,955
Lombardy	4.45	−83.44	87.89	7,535
Switzerland	0.77	−45.11	45.88	6,596

*The*

a *highest and*

b *lowest mobility values in each region during the observed period, from the COVID-19 projections of IHME^20^*.

c *The lockdown level was evaluated by the difference between the highest mobility and the lowest mobility estimated from the mobile phone mobility data*.

d *The number of available beds was obtained from COVID-19 projections of IHME^20^; the number of beds in Baden-Württemberg was calculated from the total number of beds available in Germany multiplied by the number representing the proportion of Baden-Württemberg within the total population of Germany*.

### Effects of Public Health Intervention Measures

#### Impact of Mobility (Lockdown) on the Epidemic

The policy of city lockdown involved two elements: the time of introduction of the lockdown, and the level of mobility restriction. In this study, we explored scenarios of making the lockdown date earlier or delaying it, as well as varying the level of mobility restriction after the lockdown. Our estimates do not use the dates that governments announced lockdowns, but from the actual mobility data traced through mobile phone networks.

**Figure 4:**
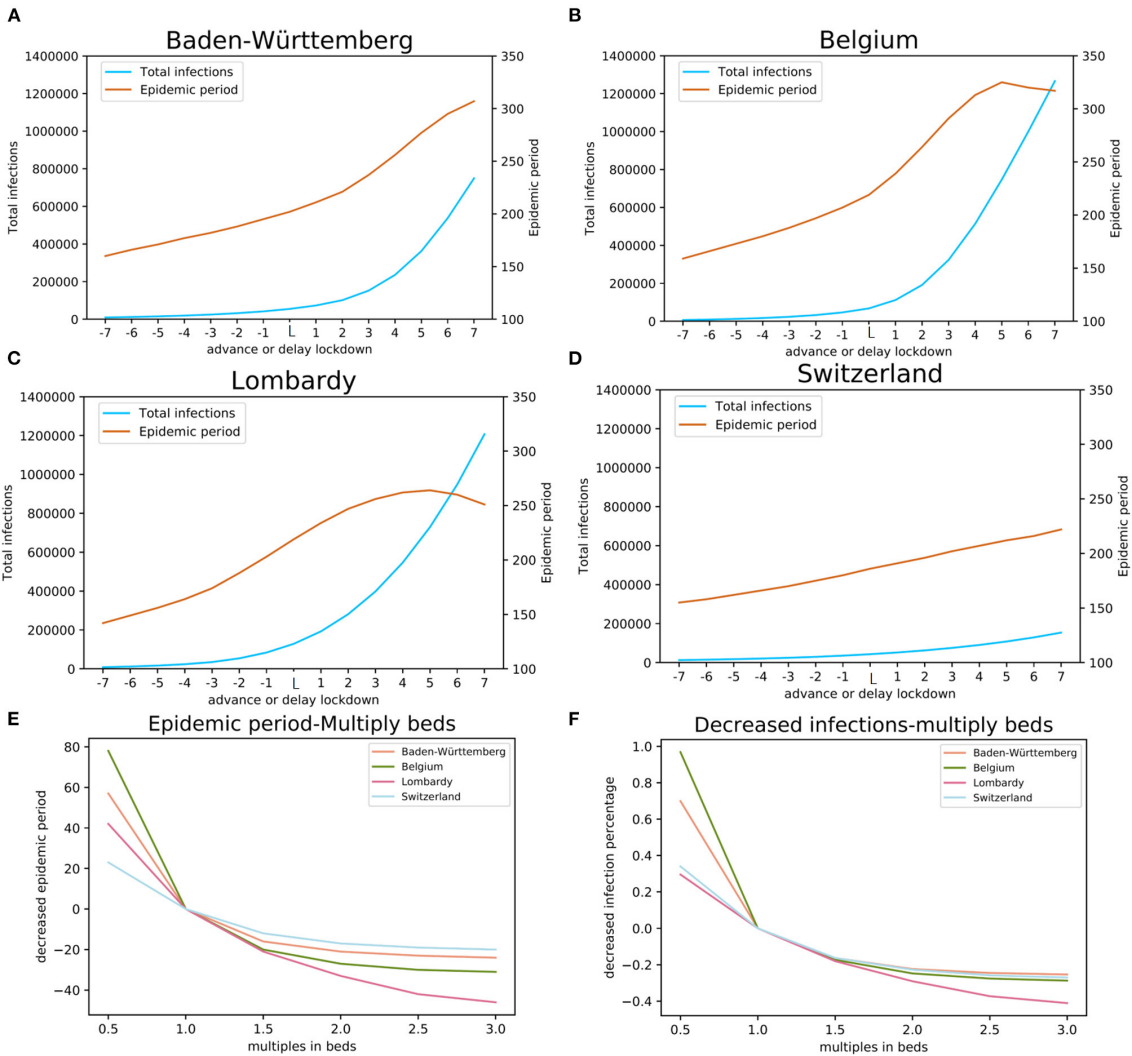
The impact of lockdown date and COVID-19 available beds. The estimates of the total number of infections and the length of epidemic period with earlier (days−7 through−1) and delayed (+1, through +7 days) lockdown relative to the actual lockdown day (day L): **(A)** Baden-Württemberg, Germany, **(B)** Belgium, **(C)** Lombardy, Italy, and **(D)** Switzerland. Estimates of the effects of multiples of available beds on the total number of infections are shown in panel **(E)** and estimates of the length of the epidemic period are shown in panel **(F)**.

We performed a simulation for the actual lockdown (“day L”) and then explored possible effects of early or delayed lockdowns. We performed 14 simulations for days −7 to −1 and days +1 to +7 relative to day L (Figures 4A–D). Modeling results suggest that early lockdown would shorten the epidemic, while the delay would prolong it. The results indicate that, maintaining the strictest level of lockdown, the 7 days earlier lockdown date of March 15 in Baden-Württemberg would have resulted in epidemic duration of 160 days (end around July 20) with 9,206 total infections, 42 days earlier than the estimated real end point of the first wave (Figure 4A). Even a one-day advance would have reduced the number of total infections by 24% (41,514 total infections) compared with the day L estimation (54,646). The simulation results suggest that one-day delay in lockdown would have resulted in 34% more infections (73,028 total infections), while the 7 days delay scenario would have a total of 749,315 infections (Figure 4A). The simulation for the four studied regions illustrated that moving the lockdown to an earlier date would significantly reduce the total number of infections and markedly shorten the time to the end point of the first wave (Figure 4). On the other hand, the delay of lockdown date would have exponentially increased the total number of infections.

The level of mobility restriction also impacts the dynamics of epidemic. The infection coefficients α and β (Table 1) are model parameters affected by the mobility: high mobility is represented by high infection coefficients, while low mobility is represented by low infection coefficients. The mobility change factor F (the “lockdown level,” Table 1) reflects the effect of mobility change to infection coefficients after the lockdown, as compared with their values before the lockdown. For the four studied regions, factor F was calculated by model fitting and has respective values of 2.55, 3.00, 3.47, and 3.85 for Switzerland, Baden-Württemberg, Belgium, and Lombardy. The spread of these values agrees well with the actual difference of mobility in the four regions (Table 3).

#### Impact of Medical Resources (Available Beds for COVID-19) on the Epidemic

We modeled the effect of local hospital capacity available for COVID-19 patients; the number of hospital beds was chosen as a proxy for such capacity (Figures 4E,F). We simulated the effects of reduced or increased number of beds for COVID-19. In our simulation, this factor had values from 0.5 to 3 with a step of 0.5. Hospital beds capacity is classified into three categories (levels): (1) sufficient, with the maximum occupancy lower than 80%, (2) heavily loaded, the maximum occupancy is higher than 80% but below the capacity, and (3) insufficient, where the occupancy demand is higher than the available beds (>100%).

The simulation results indicate that reduced available bed capacity increases the total number of infections and the increased number of beds decreases the total number of infections (Figure 4E). For Baden-Württemberg, the number of beds available for COVID-19 was sufficient, even during the most severe period. The total infections were estimated as 45,747, 42,423, 41,248, and 40,781 (bed multiple factors of 1.5, 2.0, 2.5, and 3.0, respectively). Compared with the actual situation of 54,646 total infections, increasing the number of beds would reduce the total infections by 16.3, 22.4, 24.5, and 25.4%. On the other hand, the modeling results indicate that halving the number of beds (multiplication factor of 0.5) would result in 70.0% more infections (92,841 in total). In Lombardy, where the number of available beds for COVID-19 was insufficient, increasing the bed capacity would significantly reduce the total number of infections from the actual 128,584 to 105,396, 91,199, 80,627, and 75,751 (bed multiple factors of 1.5, 2.0, 2.5, and 3.0, respectively). Our model estimated that reduction in total infections would be 18.0, 29.1, 37.3, and 41.1%, respectively. The modeling results indicate that changing the number of COVID-19 dedicated beds would change the total number of infections. An important finding from our model is, when the number of beds has already been sufficient, increasing the number of beds would result in rapidly diminishing gain and is, likely, economically not viable.

To model the effects of COVID-19 available beds we set the comparison baseline as half of the beds that were available in the health system. For each region, this number would be insufficient. The effects of the total number of COVID-19 available beds on the total number of infections and the length of epidemic period were estimated using our model with the actual number of beds, and up to three times the actual number of beds (Figures 4E,F). The results of modeling indicate that the number of COVID-19 available beds was optimized in Switzerland, Belgium, and Baden-Württemberg for managing the number of infections while for this purpose the number of beds initially available was insufficient in Lombardy (Figure 4E). On the other hand, modeling shows that the initial number of COVID-19 available beds was also optimized for the shortest duration of epidemic in Baden-Württemberg, Switzerland, and Belgium, while it was less effective in Lombardy (Figure 4F).

#### Combined Impact of the Lockdown and the Available Beds

**Figure 5:**
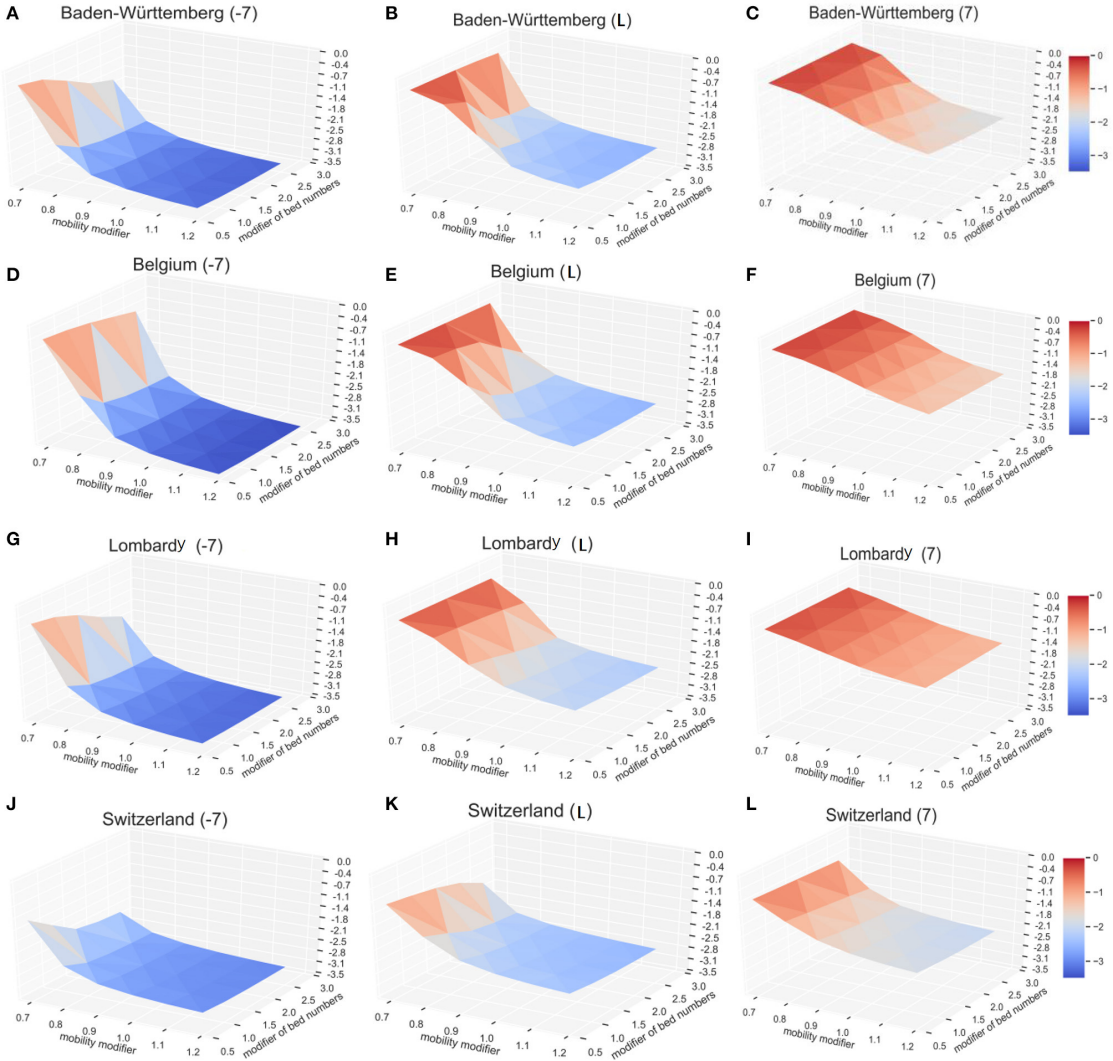
The impact of lockdown time, lockdown level, and the available beds on the number of COVID-19. The vertical axis shows relative infection rates: the normalized infection percentage after 200 days in Baden-Württemberg for **(A)** 7 days early lockdown, **(B)** actual lockdown, and **(C)** late lockdown. The corresponding results are shown for Belgium **(D–F)**, Lombardy **(G–I)**, and Switzerland **(J–L)**. For better illustration, the infection level (vertical axis) here is defined as the log_10_CDR.

To explore possible effects, we modeled three potential time points of lockdown, 7 days early (−7), the actual situation (day L), and the lockdown with 7 days delay (+7). By varying the multiplication factor of lockdown level (mobility modifier in Figure 5) from 0.7 to 1.2 (0.7 times to 1.2 times of current situation in different regions) and the COVID-19 available beds (modifier of bed numbers in Figure 5) from 0.5 to 2.5 times of current situation in different regions, we calculated the expected COVID-19 infection rates (CIR) on day 200 after the first patient (Patient 0) was identified (vertical axis—log_10_CIR in Figure 5). Modeling results indicate that the lockdown date is the primary influencing factor. For each region, the total infection rates are much lower for 7 days earlier lockdown scenario (Figure 5A) than the current situation (Figure 5B). With 7 days delay of lockdown (Figure 5C), irrespective of the bed numbers and the lockdown level, modeled infection rates are higher than the current scenario (Figure 5). Also, lockdown level significantly affects the resulting infection rate. For example, in Baden-Württemberg, the highest lockdown level (multiplication factor of 1.2) with the existing bed number (multiplication factor of 1) would reduce the infection rate at 200 days (since the start of epidemic) from 0.49 to 0.32% (Figure 5B). This would result in a 35.0% drop in the number of infections, as compared to the actual situation. The simulation results suggested that the number of infections at 200 days would drop, relative to the actual situation, by 16.3, 22.3, and 24.5% if the number beds increased by 1.5, 2.0, and 2.5 times, respectively (Figure 5B). If the lockdown level was increased to 1.2 times relative to the actual situation, even with 50% of available beds, the total infection rate at day 200 will be only 83.7% of the actual. The increase of the number of COVID-19 beds by multiplication factor of 2.5 would practically result in the control the infection at 200 days (the infection rate would drop from 0.49 to 0.27%). The lockdown date in actual situation with the lockdown factor 0.7 and beds factor of 0.5 would result in infection rate of 55.4% (Figure 5B) and increase to 56.7% (Figure 5C) with the lockdown of 7 days delay. The actual lockdown level and COVID-available beds results in infection rate at day 200 of 0.49% (Figure 5B) and 6.69% (Figure 5C) with the actual lockdown date and 7 days of delay, respectively.

Similar results were observed with the infection models in Belgium, Lombardy, and Switzerland. The solution surface for seven days earlier lockdown models in Belgium (Figure 5D), Lombardy (Figure 5G), and Switzerland (Figure 5J) show reductions of infections relative to the numbers representing actual situation (Figures 5E,H,K). The earlier lockdown models have much more pronounced improvements relative to the late lockdown (Figures 5F,I,L). Our model, as expected, shows that the early lockdown date and increased lockdown level would significantly reduce the progress of epidemic. Modeling results also suggest that sufficient medical resources—the COVID-19 available beds—help reduce the number of total infections, but their impact is lower than the impact of the lockdown level. Increasing the COVID-19 available beds by 2.5-fold (multiply by 2.5) in Baden-Württemberg would reduce the total number of infections on day 200 by 24.5%, as compared to increasing the lockdown factor by 20% (multiply by 1.2) that will reduce the number of total infections on day 200 by 35.1%.

Our observations were supported by the outcomes of epidemic in several other regions. Strict and early lockdown resulted in a rapid control of COVID-19 infection in places such as Wuhan-China (27), Denmark (28), and Norway (29). The opposite case was in Sweden that had no lockdown, and both the relative numbers of infections and deaths were larger than in the neighboring countries that have similar resources but enforced the lockdown (Denmark and Norway) (28, 29). The combination of lockdown and the rapid increase of available beds in Wuhan, China, helped achieve the effective control of the epidemic in Wuhan within 76 days (from the lockdown on January 23 to the lockdown release on April 8). Delayed increase in the number of beds in Switzerland did not markedly improve the epidemic outcomes (Figures 5J–L). While these results are intuitive, the advantage of modeling is that it provides quantitative results about expected outcomes that can be used for optimal timing of the public health measures.

#### The Impact of Lockdown Level, Bed Numbers, and the Population Age Structure on the COVID-19 Death Rate

According to our modeling results, the early lockdown, strict lockdown level, and sufficient bed numbers are essential for effective control of the epidemic. However, by investigating the current COVID-19 death rate (CDR) in 36 European countries (Supplementary Table 4), we found that the countries with high CDR (including France, Italy, and Belgium) did implement strict lockdown policies that resulted in mobility score derived from IHME (21) decreases of 92.7, 83.7, and 81.0 (absolute numbers), respectively. The population aged above 65 years in these countries are 20.4, 23.0, and 19.0%, respectively. The average mobility score in 10 countries with the highest CDR was 71.7, while the average proportion of populations aged 65 years or more was 19.45%. The corresponding data for 10 countries with the lowest CDR showed the average decrease of mobility score of 64.1, and 17.2% of their populations were older than 65 years of age.

We defined a function of COVID-19 death rate, which included the parameters of lockdown level (F), available COVID-19 beds (H), population percentage of people younger than 15 (P15), population percentage of people aged from 15 to 65 (P15–65), and the population percentage of people older than 65 (P65). By utilizing a regression model, we calculated the coefficient of each parameter: *F* = 0.003, *H* = −0.008, P15 = −0.062, P15–65 = −0.137, and p65 = 0.264. The results suggest that the population with larger proportion of P15–65 population will have fewer COVID-19 deaths, while larger P65 population will have more deaths.

### Assessment by Simulation of the Optimized Public Health Strategies

We simulated three virtual cities, named *Ytown, Mtown*, and *Otown*, with different age structures of their population. The three virtual cities were modeled to match the younger age structure of Niger (*Ytown*), average which based on the overall age structure of China (*Mtown*), and older based on the age structure of Italy (*Otown*), respectively (Supplementary Figure 1). The total population of each virtual city was set to 10 million, and the initial infection coefficients α and β were set as the average of the four simulated European regions.

The results of simulations suggest that early lockdown is the most effective policy in reducing the total number of COVID-19 infections (TNI) and total number of COVID-19 deaths (TND), regardless of the lockdown level and the number of available beds for control of COVID-19 (Supplementary Figures 2–4). The number of available beds for control of COVID-19 shows reverse-proportional effects: a larger number of beds linearly decreases the death rate (Supplementary Figure 5). The death rates are similar in the regions with younger and average age populations. The simulated death rates in these regions are approximately half of the death rates in regions with older population when other conditions are similar.

If all three virtual cities had exactly four beds per thousand people available for COVID-19 control, the lockdown policies enacted on the simulated day 24 (*T*_*SD*24_) from the day of the first patient (*T*_*SD*0_), modeling results suggest that epidemic control would be effective irrespective of the number of beds (within the range 1–4) or the level of lockdown (within the range 2–4). Early lockdown would reduce both TNI (Supplementary Figure 6) and TND (Supplementary Figures 2–4) by at least an order of magnitude (10-fold). The level of lockdown is an important consideration: in the simulation, the lockdown level 2 resulted in reduction of both TND and TNI by an order of magnitude, but both numbers kept increasing up to day 200 (*T*_*SD*200_) (Supplementary Figure 6A). The lockdown of level 3 rapidly stabilized both the TNI and TND; early lockdown (*T*_*SD*24_) resulted in TNI ~1,000 (Supplementary Figure 6B) and TND < 100 for all simulated age structures (Supplementary Figures 2D–F, 3D–F, 4D–F). For every 8 days of the lockdown delay, the TNI and TND increased approximately 10-fold (Supplementary Figures 2–4, 6).

Simulations of three cities where the lockdown factor varied from 2.0 to 4.0, increment 0.1, showed that the total number of infections was 10–100 times higher under lockdown level 2 condition than under higher (3–4) lockdown level (Supplementary Figures 2–4). The CDR (death rate of infected population) was lower for lower levels of lockdown, and conversely was higher for higher level of lockdown (Supplementary Figure 5). Four beds per 1,000 population reduced death rate in all population structures approximately by half as compared to one bed per 1,000. The simulated CDR in middle-age population (*Mtown*) showed 20–100% increase (depending on variables) as compared to the younger (*Ytown*) population. The CDR in older population (*Otown*) was approximately two to six times larger than the CDR in the young population (Supplementary Figure 5). Collectively, these results indicate complex relationships between variables (lockdown date, lockdown level, and number of beds).

**Figure 6:**
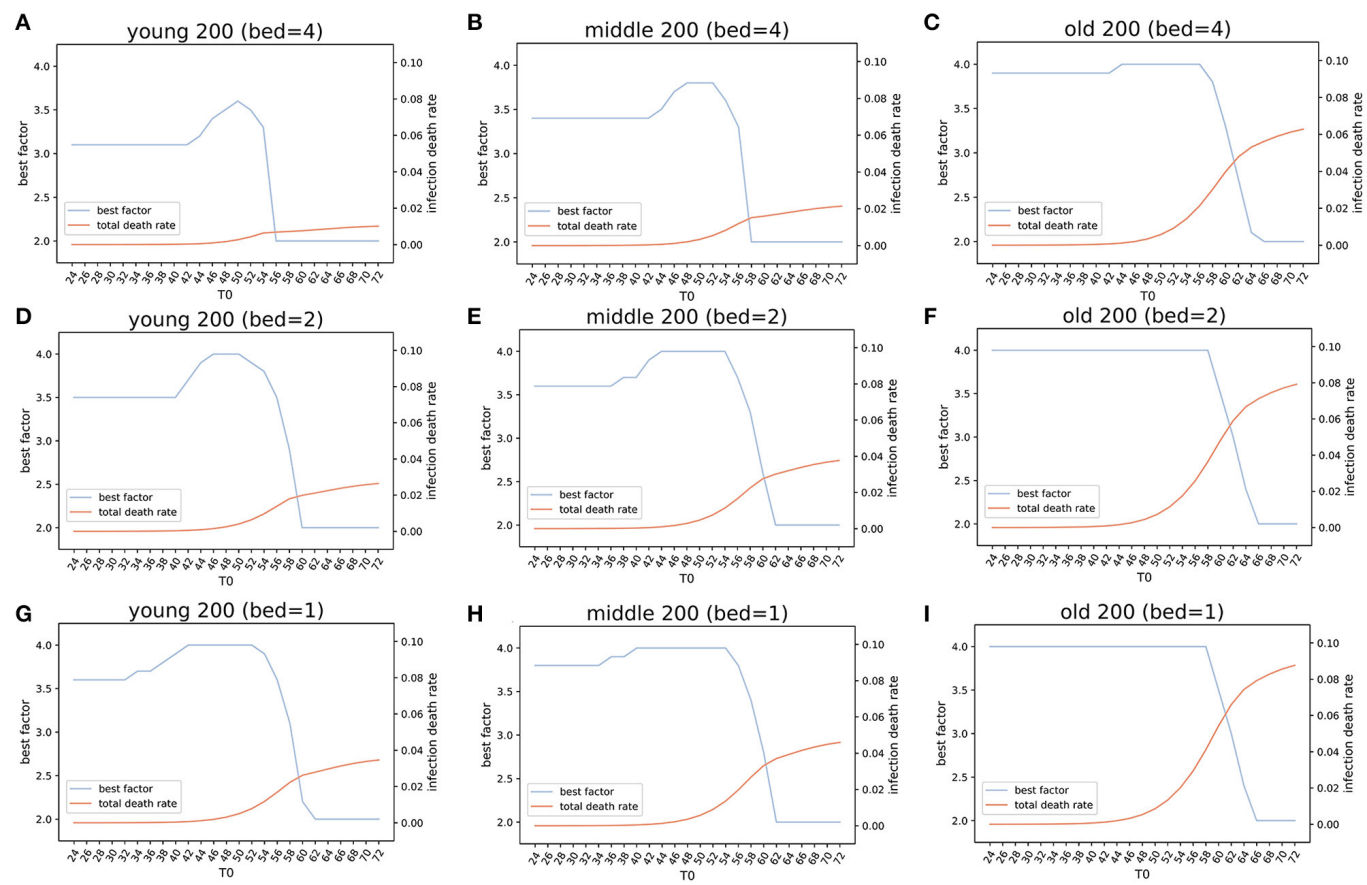
Estimation of the optimized policies in three virtual cities. Optimized lockdown factor and the corresponding death rate with different lockdown dates and four COVID-19 available beds per thousand people in three virtual cities younger, middle, and older populations). Four beds: **(A)** *Ytown*, **(B)** *Mtown*, **(C)** *Otown*. Two beds: **(D)** *Ytown*, **(E)** *Mtown*, **(F)** *Otown*. One bed: **(G)** *Ytown*, **(H)** *Mtown*, **(I)** *Otown*. The “best factor” is optimized lockdown level (factor F) that minimizes total deaths for various scenarios of lockdown date and the number of beds. The infection death rate in our model is the death rate within population that got infected at any time (total infections). The simulations indicate that the optimal policies vary between different communities.

Simulation results showed that under an early lockdown (T_0_ = 24) with the optimal lockdown level (*Ytown* = 3.1, *Mtown* = 3.4, and *Otown* = 3.9), the overall death rate in populations (PDR) for all three virtual cities were < 0.001% (PDR_*Ytown*_ = 0.00039%, PDR_*Mtown*_ = 0.00053%, and PDR_*Otown*_ = 0.00096%). The corresponding TND values after 200 days (TND_SD200_) were 39 for *Ytown*, 53 for *Mtown*, and 96 for *Otown* (Figures 6A–C). Considering that on day 24, the number of observed infections was only 105, it is unlikely that, at this point, the local authorities would notice the epidemic if it was the first epidemic outbreak (like COVID-19 outbreaks in Wuhan, China, or Lombardy, Italy). However, if local authorities were on alert, due to knowledge of the ongoing epidemics in other regions, like in Australia, early responses appear to be viable options. Our simulations suggested that if the lockdown is delayed for 8, 16, and 24 days, the TND_SD200_ number in *Ytown* will quickly increase from 39 (T_SD24_) to 264 (T_SD32_), 1773 (T_SD40_), and 15,962 (T_SD48_).

For *Ytown*, if COVID-19 available beds were four per 1,000, and the city lockdown happened between day T_SD24_ and T_SD42_, the total death rate would range from 0.00039 to 0.029%. The TND would be between 39 and 2,873 for the optimized lockdown of 3.1 (Figure 6A). If the local authority started lockdown T_SD44_ and T_SD54_, the TND would increase sharply, and the optimized lockdown factor would change from 3.2 to 3.6. These results reflect the need for dynamic management of public health policies in response to different situations. During the lockdown starting between T_SD44_ and T_SD54_, the TND would range from 4,950 to 63,629. However, if the local authority provided no lockdown policies before day 56, the later lockdown policies will have little effect. This scenario indicates that more than 50% of the population will be infected, and the overall deaths will be close to 1% of the total population (Figure 6A). The early lockdown response has proven to be effective in control of the second wave of COVID-19 in Victoria, Australia (lockdown from August 2 to September 6, which was gradually easing until October 26). Similar successes in the control of COVID-19 epidemics with early lockdowns were reported in Greece (30) and South Africa (31).

The results of simulations in *Mtown* were similar to the *Ytown* results. When T0 ranged from 24 to 42, the optimal lockdown factor was 3.4, with total death rate slowly increasing. The overall simulated death number was between 53 (day 24) and 3,908 (day 42). The second stage was for T0 between 44 and 56, with the optimal factor changed from 3.5 to 3.8 and the overall deaths from 6,586 to 119,513. After 58 days, the optimized policy is pursuing herd immunity, which will result in 1.5–2.0% deaths within the whole population (Figure 6B). Interestingly, the optimized lockdown factor is different for three virtual cities. For *Otown* (city with >23% of the aged population), the best policy is strict lockdown of the city with the lockdown factor of *F* = 3.9, close to the upper limit in our simulation (Figure 6C). However, for *Ytown* and *Mtown*, the best policy may not be the total lockdown of the city. Our estimation showed that the lockdown factors between *F* = 3.1 and *F* = 3.4 will result in the lowest total death rate. This indicates that different public health strategies are appropriate for cities with different age structures. To reduce the CDR for aged people, an early strict lockdown policy is needed. In fact, in *Otown*, the lockdown level of 4 will be the optimal policy when the lockdown is not announced early (Figure 6C). If herd immunity policy is pursued (no lockdown) in *Otown*, without any reduction in mobility, the overall death will increase to 627,867 and the death rate will exceed 6% even if the beds are sufficient (four per thousand).

The number of available beds is a modifier of best lockdown level in simulated scenarios. If the number of available beds for COVID-19 is halved (2 per thousand), the optimized factor of lockdown would be increased from *F* = 3.1 to *F* = 3.5 for *Ytown* (Figure 6D), from *F* = 3.4 to *F* = 3.6 for *Mtown* (Figure 6E), and from *F* = 3.9 to *F* = 4.0 for *Otown* (Figure 6F), respectively. When the number of beds is reduced to one quarter (one per thousand), the optimized lockdown factor would increase to *F* = 3.6 for *Ytown* (Figure 6G), *F* = 3.8 for *Mtown* (Figure 6H), and *F* = 4.0 for *Otown* (Figure 6I). Moreover, reducing the number of beds will result in more deaths for any lockdown date irrespective of the adjustment of the lockdown factor.

Overall, our results suggest that (1) reducing the social distance (lockdown) at the early stage is the most effective policy to reduce total infections, and (2) the optimized level of lockdown differs for cities with different age structures. For an aged society, strict lockdown appears to be more effective in reducing the CDR. For younger societies, relatively loose lockdown level (around *F* = 3 to *F* = 3.4) may minimize the total death rate. (3) The increase of the number of COVID-19 available beds strongly impacts both the infection rate and the total death rate when numbers are insufficient; when beds are sufficient, the improvements in infection rate and CDR are modest (diminishing returns). The proposed SEIR(MH) model can quantify the combined impact of multiple public health interventions in populations that have different characteristics and simulations have shown excellent concordance with the actual situations in studied regions. This model has a potential to assist in designing optimized public health interventions in regions that have different sociodemographic properties.

## Discussion

The global pandemic of COVID-19 is a huge public health issue for human society. During the epidemic period, adequate nowcasting (estimating the current status) and forecasting (predicting future status) are crucial for public health planning and epidemic control (5, 28). We constructed a real-time status dynamic SEIR(MH) model to estimate the epidemic in local geographic areas. By adding the parameters of status M and H to a traditional SEIR model, we accurately modeled COVID-19 epidemics for four European regions. Our model allows quantification of the lockdown measures using mobility as proxy. Also, we could quantify the effects of available bed capacity. The quantification allows forecasting of the effects of public health measures and optimizing their impact under different constraints. The SEIR(MH) model could simulate the effects of public health policies in isolation or in combination, such as assessing the effects of (1) the date of lockdown measure, (2) the level of lockdown, (3) the number of dedicated beds, and (4) the effect of population age structure. The SEIR(MH) model can help rapidly assess the possible effects of complex combinations of public health measures for the epidemic control.

The timing of mobility restriction (lockdown) is the most important public health measure for the control of an epidemic that has characteristics of COVID-19. The lockdown at early stage will help quickly end the epidemic with significantly reduced total infections and death numbers. The analysis of data from 184 countries indicated that, on average, better control of COVID-19 epidemic correlated with earlier lockdowns (32). We defined four levels of lockdown: basic (*F* = 1), low (*F* = 2), moderate (*F* = 3), and strict (*F* = 4). Our simulation results suggest that the lockdown that starts only one week earlier than the lockdown dates observed for COVID-19 would end the epidemic 42 days earlier than the current situation and reduce the number of total infections in a region with over 10 million populations such as Baden-Württemberg, Germany, by more than 80%. On the other hand, 7 days delay would lead to 16-fold increase in total infections than the observed situation. Based on our estimates, Belgium responded most quickly in 31 days after the potential patient 0, followed by Italy (38 days considering the 7 days modification) and Germany (39 days). Switzerland did not announce the lockdown policy in early stages and delayed the lockdown after almost two months of the estimated patient 0. The decision of lockdown in a region with 10 million populations is not an easy decision, since the lockdown will significantly affect the daily activities of the citizens, affect economic development, and create other health problems due to reduced access to regular health care, among others. It is not feasible to lock down a city or a region when only a few cases are discovered. However, the epidemic like COVID-19 transmits rapidly at the early stage; therefore, it is easy to miss the best window of opportunity for epidemic prevention and control. Potential utility of such models is high because regional health authorities can easily get informed from the regions that experienced early outbreaks, such as Wuhan in China and Lombardy in Italy.

The lockdowns also increase the pressure on local medical resources. By using the COVID-19 available beds as proxy, our model illustrated the effect of increasing medical resources. In bed-sufficient regions such as Baden-Württemberg, Germany, the increase of the number of COVID-19 available beds will slightly decrease the total infections. Our estimation for Baden-Württemberg, Germany, suggested that doubling COVID-19 available beds would decrease 22.4% of infections, while tripling the number of COVID-19 available beds would result in 25.4% decrease of total infections. In bed-insufficient regions, such as Lombardy, Italy, doubling or tripling the current available beds would result in the decrease of total infections by 29.1 and 41.1%, respectively.

The lockdown level for epidemic control is important but, interestingly, our modeling indicates that strict lockdown is not always the best solution for controlling epidemics. Our model has suggested that strict lockdown (*F* = 3.8 to *F* = 4.0) is effective only in regions with older population. For populations that have middle or younger age structure, moderate lockdown measures (*F* = 3.0 to *F* = 3.4) may produce better epidemiological outcomes. Obviously, more strict restrictions will lead to larger social distance and reduce the number of total infections. However, the total lockdown may increase pressure on local medical infrastructure including rapidly growing demands for hospitalization and shortage of medical staff and medical supplies, which may lead to increased death rate from other causes. In the first wave of COVID-19, regions with higher death rate such as France, Italy, and Belgium imposed high-level lockdown with a very high mobility decrease (21). The age structure of population is important; populations with older age structure have shown a higher COVID-19 death rate (24, 33). According to our simulation of three virtual cities, stricter lockdown policies, around 3.8 to 4.0, are required to decrease the total COVID-19 death rate in societies with older age structure. On the other hand, looser lockdown policies, around 3.2–3.4, may be preferred for populations with lower or middle age structures. The analysis of data from 184 countries (32) suggested that partial lockdowns may be as effective in controlling the epidemic as strict lockdowns. The advantage of the SEIR(MH) model is that it offers not only qualitative assessment but it also produces quantitative projections that can be used for comparative analysis of the effects of combined public health interventions.

Most of the European regions released the lockdown and now are experiencing the second wave of the epidemic (21). The second COVID-19 wave has different characteristics, with larger number of infections, lower death rates, different demographics of epidemics, and the availability of vaccines. We considered only the first wave of COVID-19 for our modeling.

Our modeling indicates that the relationships between public health measures and the epidemic outcomes (including the length of epidemic period and total number of infections) are complex and depend on the population behavior that can be captured in mobility and other geo-social data (34). A note of caution is that these results should be used only for better understanding of the effects of specific public health measures (level, start time, and the duration of lockdown, as well as the management of the number of available beds) on the dynamics and the direct outcomes of COVID-19 epidemics. The lockdowns and rearranging the bed capacity for the control of an epidemic will have a broader range of socio-economic and medical consequences that need to be considered in parallel with analyses that focus purely on the epidemic.

While studied regions are adjacent and have similar population and relative level of economic development, their key underlying public health parameters are very different. This is best observed in the differences in infection parameters, mobility factor F (Table 1), and mobility levels before and after the lockdown (Table 3). The mobility factor F and the level of mobility decrease are related. For example, the mobility factor of 3.0 means the pre-lockdown infection coefficients α_pre_ and β_pre_ are three times larger than the post-lockdown infection coefficients α and β. Higher mobility factor F means stricter lockdown level. On the other hand, the mobility level was calculated from the observed mobility data in IHME. The difference between the highest mobility before lockdown and the lowest mobility after lockdown were used to calculate the level of mobility decrease. The mobility factors were estimated by parameter fitting, while the lockdown levels were calculated from the observed data. In the four studied regions, the real lockdown levels were Lombardy (87.89) > Belgium (81.05) > Baden-Württemberg (69.65) > Switzerland (45.88). These data were consistent with the estimated values of F: Lombardy (3.85) > Belgium (3.47) > Baden-Württemberg (3.00) > Switzerland (2.55). Methods of reporting COVID-19 cases and approaches to protecting elderly are also different between the regions (35). Therefore, the absolute numbers of reported cases are not directly comparable, but the shapes of the infection curves indicate the actual dynamics of epidemics in studied regions. Our model has demonstrated robustness since it produced infection curves that closely resemble the actual reported numbers, where all modeled infection curves show good agreement with the actual data. The COVID-19 infection curves are non-linear and asymmetric, showing a rapid exponential growth that reaches the peak followed by a delayed reduction in new cases, with a long right tail spreading throughout the summer, never reaching zero.

### Limitations

The issues that affect the relevancy and accuracy, or limitations, of the model are data issues and model issues. The data issues include the complexity and hierarchical nature of real-world processes that generate data, fuzziness of data, biases and potential misconceptions in data, and the noise and errors in data (36). Mathematical models are simplifications of real-life systems and are based on assumptions that approximate real-life situations (21). Considering the extremely complex nature of epidemics/pandemics, any epidemic model will be a simplification of the real situation that may vary from one region to another. Mathematical modeling requires compromises; the results of modeling must be reasonably accurate, but modeling must also be computationally viable. To make our model realistic, data were smoothed, and the model parameters were fitted to data. Necessary corrections were made to the model, when discrepancies between the model output and the actual data were observed. We considered model adjustments and collected additional evidence to justify these changes. The simplifying assumptions of the regional SEIR(MH) model include considering the epidemic in geographic areas that are isolated and our model assumes that the infections rate in each geographic area is divided into two stages, before the lockdown and after the lockdown, with constant infection rate throughout the first stage of epidemic, and reduced infection rate, another constant, throughout the second stage of epidemic. While these limitations are a modeling concern, the conclusions derived from the results of simulations are consistent with the observed data across different countries (21, 22). Irrespective of the conditions specific for different countries, the SEIR(MH) model has demonstrated it is robust and it enables the analysis of outcomes of public health measures. This strategy needs to be combined with vaccination because early lockdown slows down the development of herd immunity.

## Conclusions

In general, as the simplification of real-life systems, the mathematical models could approximate real-life situations based on reasonable assumptions. In this study, we extended the conventional SEIR model by adding the parameters that define public lockdown and the the number of dedicated hospital beds to simulate the real-life situations such as lockdown policies or construction of temporary hospitals in measured regions. Further, by performing simulations on virtual cities with different age structure, our model could provide optimized policy combinations by setting the total infections and COVID-19 related death rate as goal. The robustness of the SEIR(MH) model illustrated the utility of this model to analysis the outcomes of different public health measures.

## Data Availability Statement

The original contributions presented in the study are included in the article/Supplementary Material, further inquiries can be directed to the corresponding author/s.

## Author Contributions

TQ and VB collected the data, designed the study, performed simulations, interpreted the results, and co-supervise the whole project. HX designed and implemented the model and performed parameter fitting. All authors contributed to the article and approved the submitted version.

## Funding

This work is supported in part by grants from the National Natural Science Foundation of China (31900483) and Shanghai Sailing program (19YF1441100).

## Conflict of Interest

The authors declare that the research was conducted in the absence of any commercial or financial relationships that could be construed as a potential conflict of interest.

## Publisher's Note

All claims expressed in this article are solely those of the authors and do not necessarily represent those of their affiliated organizations, or those of the publisher, the editors and the reviewers. Any product that may be evaluated in this article, or claim that may be made by its manufacturer, is not guaranteed or endorsed by the publisher.
