# Supplementary material for: Estimating the Effects of Public Health Measures by SEIR(MH) Model of COVID-19 Epidemic in Local Geographic Areas
Source: Front Public Health. 2022 Jan 4;9:728525. doi: 10.3389/fpubh.2021.728525 (PMC8764356; doi:10.3389/fpubh.2021.728525)
Supplement: Supplementary file 1 [file Data_Sheet_1.pdf]

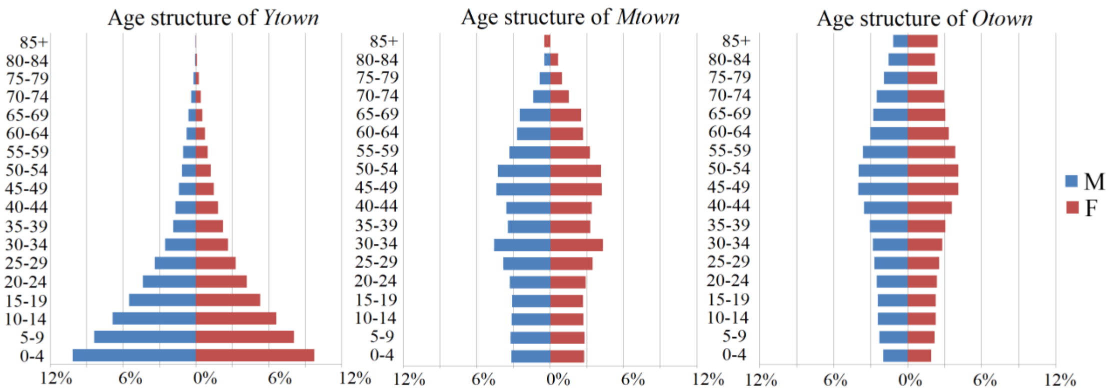

Supplementary Figure 1. Age structure of three virtual cities. Percentages of the total population (horizontal axis) for age groups (vertical axis) in *Ytown*, *Mtown* and *Otown* are shown in panels a, b and c.

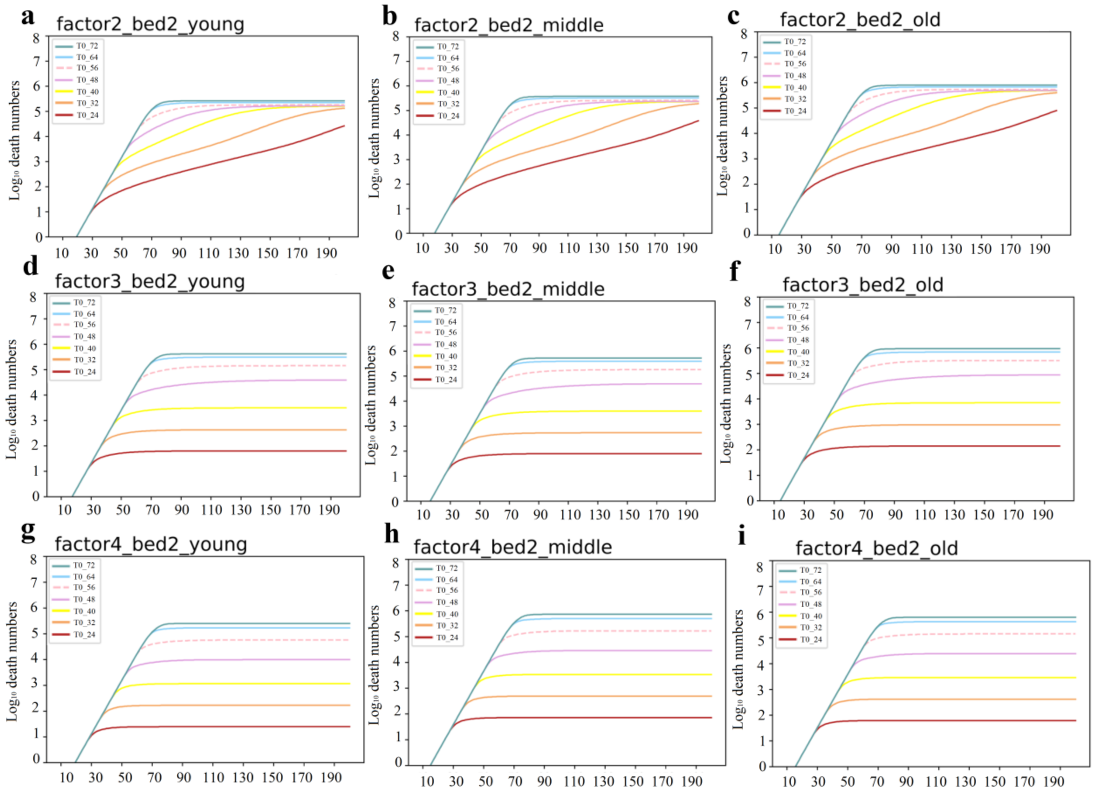

Supplementary Figure 2. Number of deaths simulated in three virtual cities using SEIR(MH) model. The vertical axis shows logarithmic value of the number of deaths (1 stands for 10, 2 for 100, etc.). The horizontal axis shows number of days between the first patient and the lockdown date (T0_NN, where NN range is from 24 to 72, T_{SDNN} in the main text). The number of beds was fixed to 4 per 10000 population. The values for *Ytown* are shown in panels a, d, and e (lockdown factors 2, 3, and 4). The corresponding values for *Mtown* are shown in panels b, e, and h and for *Otown* in panels c, f, and i. The COVID-19 beds factor is defined as 2 in this part of the study.

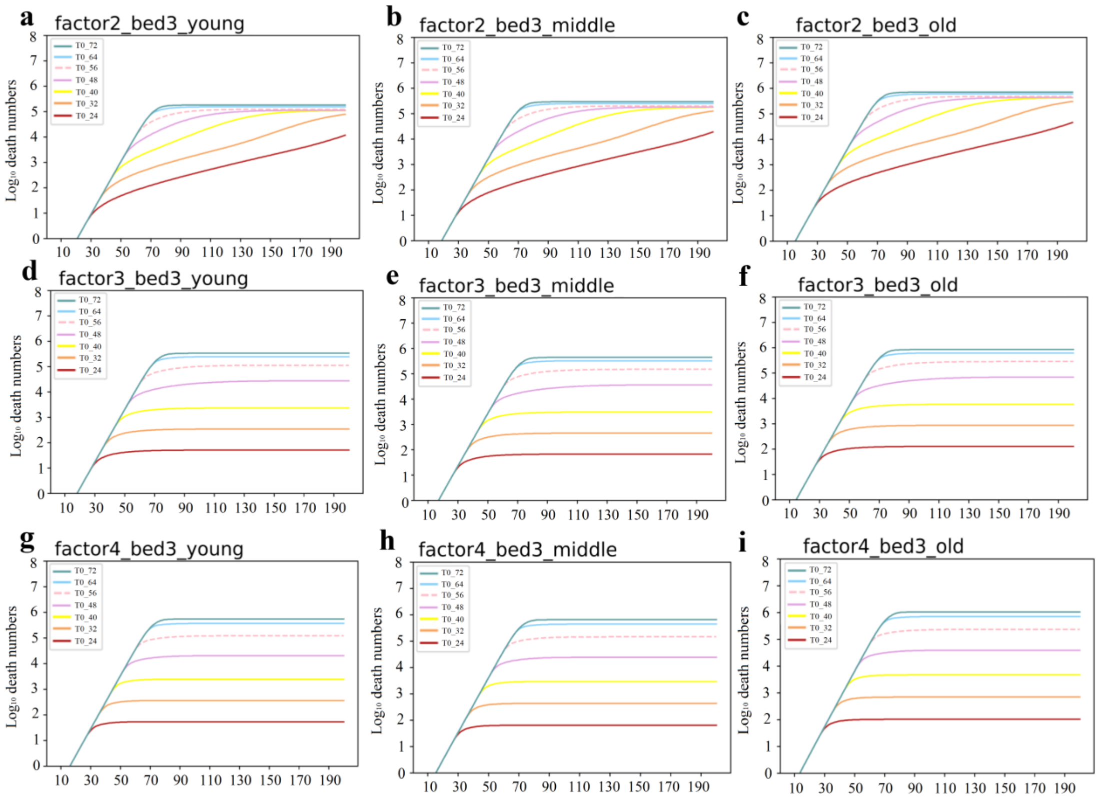

Supplementary Figure 3. Number of deaths simulated in three virtual cities using our SEIR(MH) model. The vertical axis shows logarithmic value of the number of deaths (1 stands for 10, 2 for 100, *etc*.). The horizontal axis shows number of days between the first patient and the lockdown date (T0_NN, where NN range is from 24 to 72, $T_{SD\mathrm{NN}}$ in the main text). The number of beds was fixed to 4 per 10000 population in this simulation. The values for *Ytown* are shown in panels a, d, and e (lockdown factors 2, 3, and 4). The corresponding values for *Mtown* are shown in panels b, e, and h and for *Otown* in panels c, f, and i. The COVID-19 beds factor is defined as 3 in here.

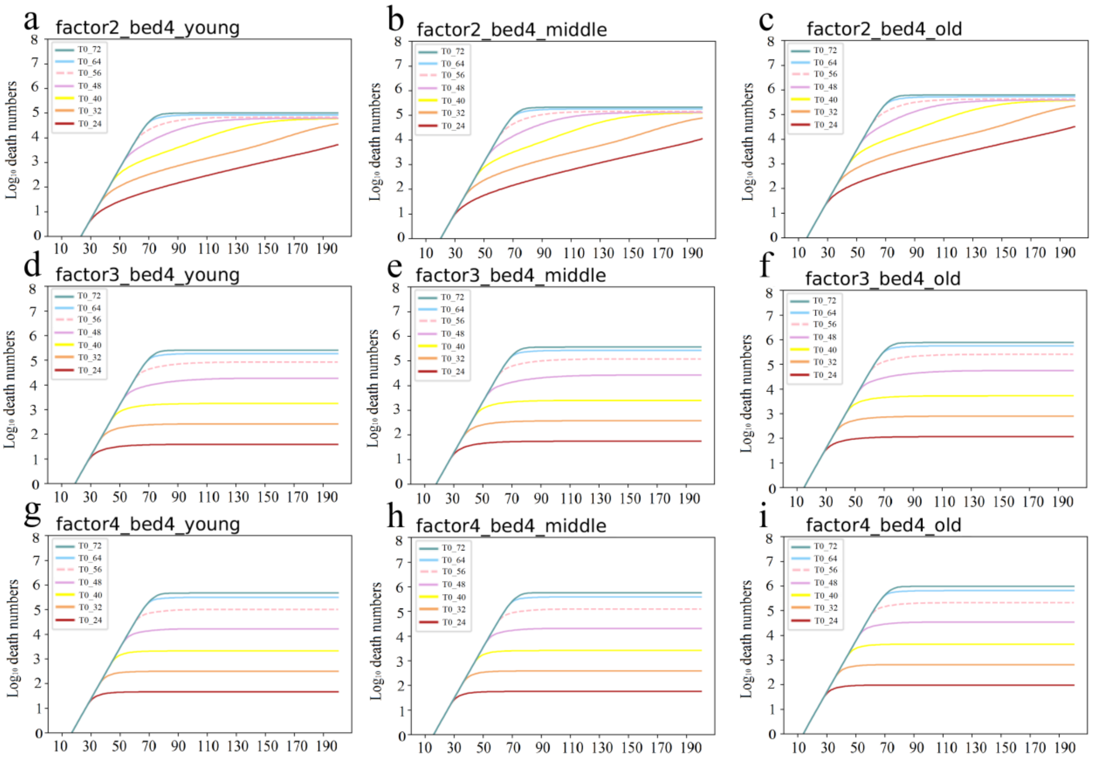

Supplementary Figure 4. Number of deaths simulated in three virtual cities using SEIR(MH) model. The vertical axis shows logarithmic value of the number of deaths (1 stands for 10, 2 for 100, etc.). The horizontal axis shows number of days between the first patient and the lockdown date (T0_NN, where NN range is from 24 to 72, T_{SDNN} in the main text). The number of beds was fixed to 4 per 10000 population in this simulation. The values for Ytown are shown in panels a, d, and e (lockdown factors 2, 3, and 4). The corresponding values for Mtown are shown in panels b,e, and h and for Otown in panels c, f, and i. The COVID-19 beds factor is defined as 4 in here.

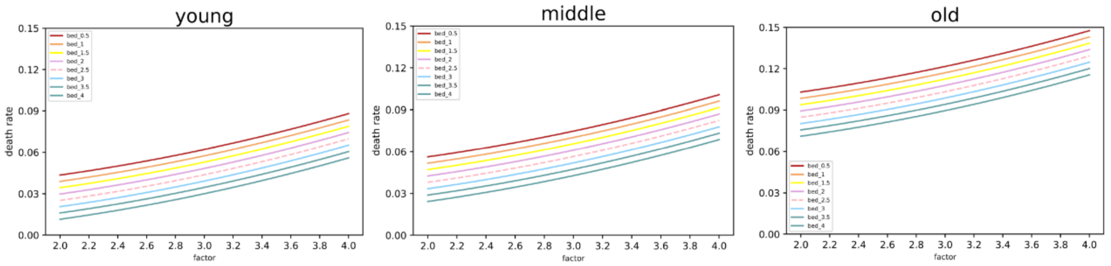

Supplementary Figure 5. COVID-19 death rate simulated in three virtual cities. The horizontal axis shows the lockdown level represented by factor (2-4), the vertical shows the death rate under different lockdown levels and COVID-19 available beds (0.5 per thousand to 4 per thousand).

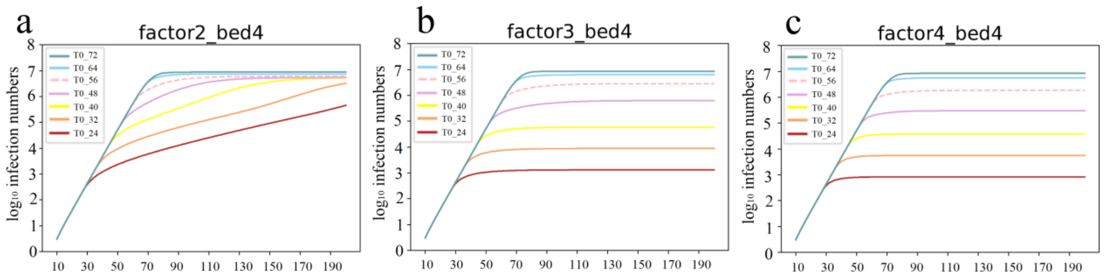

Supplementary Figure 6. Number of infections simulated in three virtual cities using the SEIR(MH) model. The vertical axis shows log values of infections (1 stands for 10, 2 for 100, *etc*.). When variables are the same, the infection numbers TNI are the same for each of the three virtual cities.

Supplementary Tables

Supplementary Table 1. Mobility and COVID-19 available beds for four European regions[a].

Country	Maximal mobility	Minimal mobility	Lockdown level	Bed	Bed per thousand
**Switzerland**	0.772	-45.110	45.882	6596.000	0.769
**Germany/B-W**	14.131	-55.546	69.677	18114	1.636
**Belgium**	3.429	-77.303	81.048	12955.000	1.128
**Italy/Lombardy**	5.332	-83.416	88.748	7535.000	0.757

[a]The mobility and bed numbers are derived from the COVID-19 projections of IHME (https://covid19.healthdata.org/).

Supplementary Table 2. Parameters setting of three virtual cities. The single value indicates that this parameters is the same for three cities, indicated by "(same)".

Variable	Ytown	Mtown	Otown
**Population**		10,000,000 (same)	
**α before lockdown**		3.7 (same)	
**β before lockdown**		4.5 (same)	
**Range of Mobility change factor**		[2.0, 4.0] (same)	
**Mobility change factor increase**		0.1 (same)	
**Initial COVID-19 available beds**		10,000 (same)	
**Range of bed change factor**		[0.5, 4.0] (same)	
**Bed change factor increase**		0.5 (same)	
**Region of days between first patient to lockdown**		[24,72] (same)	
**Step of days between first patient to lockdown**		2 (same)	
**age <15**	0.498	0.178	0.132
**15≤age<65**	0.476	0.707	0.638
**65≤age**	0.026	0.115	0.230

Supplementary Table 3. Age structure in four regions including Belgium, Baden-Württemberg (Germany), Lombardy (Italy), and Switzerland[a].

Country	<15	15-65	>65
**Italy**	0.13	0.64	0.23
**Belgium**	0.17	0.64	0.19
**Switzerland**	0.15	0.66	0.19
**Germany**	0.14	0.65	0.21

[a]The population data of each country were collected from the World Bank at https://data.worldbank.org/indicator/SP.POP.TOTL, 2019.

Supplementary Table 4. Population, infections, deaths and death rate of 36 European countries[a].

Country	Population[a]	Infections[b]	Deaths[b]	Death rate[c]
**Russia**	144373535	932493	15872	0.017
**Germany**	83132799	224014	9232	0.041
**France**	67059887	205380	30296	0.148
**United Kingdom**	66834405	319201	41369	0.130
**Italy**	60297396	254235	35400	0.139
**Spain**	47076781	342813	28617	0.083
**Ukraine**	44385155	94436	2116	0.022

**Poland**	37970874	57279	1885	0.033
**Romania**	19356544	71194	3029	0.043
**Netherlands**	17332850	63424	6163	0.097
**Belgium**	11484055	78441	9944	0.127
**Greece**	10716322	7222	230	0.032
**Czechia**	10669709	20202	399	0.020
**Sweden**	10285453	85045	5787	0.068
**Portugal**	10269417	54234	1779	0.033
**Hungary**	9769949	4970	609	0.123
**Belarus**	9466856	69589	613	0.009
**Austria**	8877067	23717	729	0.031
**Switzerland**	8574832	38156	1715	0.045
**Bulgaria**	6975761	14500	512	0.035
**Serbia**	6944975	29782	677	0.023
**Denmark**	5818553	15740	621	0.039
**Finland**	5520314	7752	334	0.043
**Slovakia**	5454073	2907	31	0.011
**Norway**	5347896	10004	261	0.026
**Ireland**	4941444	27313	1774	0.065
**Croatia**	4067500	6656	166	0.025
**Bosnia and Herzegovina**	3301000	16137	481	0.030
**Lithuania**	2786844	2436	81	0.033
**Moldova**	2657637	30377	908	0.030
**Slovenia**	2087946	2438	124	0.051
**Latvia**	1912789	1323	32	0.024
**Estonia**	1326590	2192	63	0.029
**Luxembourg**	619896	7469	124	0.017
**Malta**	502653	1274	9	0.007
**Iceland**	361313	2014	10	0.005

[a]The population data of each country were collected from the World Bank at https://data.worldbank.org/indicator/SP.POP.TOTL, updated in 2019. [b]The COVID-19 infections and deaths data of each country were collected from World Health Organization at https://covid19.who.int/table, updated on August 18, 2020. [c]The death rate of each country was calculated as the deaths/infections.
